# Knowledge of Medicinal Plants for Children Diseases in the Environs of District Bannu, Khyber Pakhtoonkhwa (KPK)

**DOI:** 10.3389/fphar.2017.00430

**Published:** 2017-07-17

**Authors:** Shabnam Shaheen, Safdar Abbas, Javid Hussain, Fazal Mabood, Muhammad Umair, Maroof Ali, Mushtaq Ahmad, Muhammad Zafar, Umar Farooq, Ajmal Khan

**Affiliations:** ^1^Department of Plant Sciences, Quaid-i-Azam University Islamabad, Pakistan; ^2^Khyber Pakhtunkhwa Southern Area Development Project Lakki Marwat (Ministry of Planning and Development Department KPK) Khyber Pakhtunkhwa, Pakistan; ^3^Department of Biochemistry, Faculty of Biological Sciences, Quaid-i-Azam University Islamabad, Pakistan; ^4^Department of Biological Sciences and Chemistry, College of Arts and Sciences, University of Nizwa Nizwa, Oman; ^5^Department of Chemistry, COMSATS Institute of Information Technology Abbottabad, Pakistan; ^6^UoN Chair of Oman Medicinal Plants and Marine Products, University of Nizwa Nizwa, Oman

**Keywords:** Bannu, Khyber Pakhtunkhwa (KPK), ethnomedicinal plants, children disorders, use value, factor of informant consensus (FIC)

## Abstract

Medicinal plants are important treasures for the treatment of different types of diseases. Current study provides significant ethnopharmacological information, both qualitative and quantitative on medical plants related to children disorders from district Bannu, Khyber Pakhtunkhwa (KPK) province of Pakistan. The information gathered was quantitatively analyzed using informant consensus factor, relative frequency of citation and use value method to establish a baseline data for more comprehensive investigations of bioactive compounds of indigenous medicinal plants specifically related to children disorders. To best of our knowledge it is first attempt to document ethno-botanical information of medicinal plants using quantitative approaches. Total of 130 informants were interviewed using questionnaire conducted during 2014–2016 to identify the preparations and uses of the medicinal plants for children diseases treatment. A total of 55 species of flowering plants belonging to 49 genera and 32 families were used as ethno-medicines in the study area. The largest number of specie belong to Leguminosae and Cucurbitaceae families (4 species each) followed by Apiaceae, Moraceae, Poaceae, Rosaceae, and Solanaceae (3 species each). In addition leaves and fruits are most used parts (28%), herbs are most used life form (47%), decoction method were used for administration (27%), and oral ingestion was the main used route of application (68.5%). The highest use value was reported for species *Momordica charantia* and *Raphnus sativus* (1 for each) and highest Informant Consensus Factor was observed for cardiovascular and rheumatic diseases categories (0.5 for each). Most of the species in the present study were used to cure gastrointestinal diseases (39 species). The results of present study revealed the importance of medicinal plant species and their significant role in the health care of the inhabitants in the present area. The people of Bannu own high traditional knowledge related to children diseases. In conclusion we recommend giving priority for further phytochemical investigation to plants that scored highest FIC, UV values, as such values could be considered as good indicator of prospective plants for discovering new drugs and attract future generations toward traditional healing practices.

## Introduction

Children are the most susceptible to various types of viral diseases and infectious due to low immune system. There are many important diseases which are common in children worldwide such as gastrointestinal, respiratory, urinary, kidney disorders, liver, ear nose throat disease (ENT), eye infection, and dental anomalies. Immune system diseases as a result of nutrition deficiency are the key element for child diseases. Evidence of the epidemic nature of diarrhea and malnutrition in children can be found in research from the Indian subcontinent, Asia, Africa, and South America. For example, in rural Bangladesh, the occurrence of diarrheal illness under the age of 5 years has been approximated to be 12.8 days per 100 child-days, which indicates that each child spent 46 days per year with diarrhea (Black et al., 1982). Other studies from Indian villages in children under 5 years of age showed varied incidences of 0.7 episodes per child per year (Bhan et al., 1986) to 2.2 episodes per child per year (Oyejide and Fagbami, 1988). Most of the plant species are used for the treatment of anemia, malaria and diarrhea among children in different parts of the world. The nutrition supplements plants are mostly used in children diseases management such as *Acacia seyal Delile, Albizia coriaria Welw., Dicliptera laxata C.B.Clarke, Kalanchoe densiflora Rolfe, Persea Americana Mill*. and *Vernonia amygdalina Delile* having different micronutrients and macronutrients except phosphorus and sodium. In different countries acute gastroenteritis causes dehydration in different aged children's and the main agents of acute gastroenteritis were Norovirus and Rotavirus. Norovirus was the most detected agent in infants and young children (24 months of age) (Bicer et al., 2012). Of the estimated total of 190 million underweight children, about 120 million live in the four most populated Asian countries, which are China, India, Pakistan, and Bangladesh (UNICEF, 1993). In Pakistan, infants under 1 year old are extremely susceptible to morbidity and mortality from diarrhea; the reported death rate is 200,000 annually (Dialogue on Diarrhea, 1989).

It is well documented that herbal medicine provide a promising source of anti-diarrheal drugs and potentially valuable antimicrobial plant compounds or their extracts preferably display activity against a wide range of microorganisms (Gram et al., 2002). The commonest avoidable, cause of infant deaths are the respiratory disease (Jones, 1997). In the rural communities, wounds arising from bruises, cuts and scratches are sometimes untreated at the initial stages, which is also common problem. In most cases such wounds become septic and inflamed before they are brought to the attention of parents, who might treat such wounds in a traditional way using plant materials or seek the advice of an herbalist (Grierson and Afolayan, 1999). According to the World Health Organization (WHO), about 4 billion people in developing countries not only believe in the healing properties of plant species but also use them regularly (Rai et al., 2000).

Khan et al. (2013, 2015) studied the ethno-botany of the Bannu District, but their study was not specific to a particular disease. Moreover, the data was not quantitative analyzed for a particular disease. There are gaps in ethno-botanical knowledge in this region of Khyber Pakhtunkhwa (KPK), Pakistan that have to be explored. Therefore, there was a need to explore the remaining territory in this district, using a quantitative approach for a particular disease. The present study aimed (i) to investigate and document plant species that are used for the treatment and prevention of various health problems related to child diseases; (ii) to document traditional recipes from medicinal plants, including methods of preparation, dosage, and modes of administration; (iii) to select candidate medicinal plant species of high priority for phytochemical and pharmacological analyses in our subsequent studies; and (iv) to inform the community about the diversity and conservation of medicinal plants.

## Materials and methods

### Description of the study area

Bannu city is situated 32°16′N and 33°5′N and between 70°23′E and 71°16′E at an altitude of 371 m above sea level with an area of 1,670 square miles. It borders North Waziristan to the northwest, Karak to the northeast, Lakki Marwat to the southeast, and South Waziristan to the southwest. The population is 100% ethnic Pashtun. The main tribes are: Bannuchi, Wazir, Mehsud, Dawar, Marwat, and Bangash.

**Figure 1:**
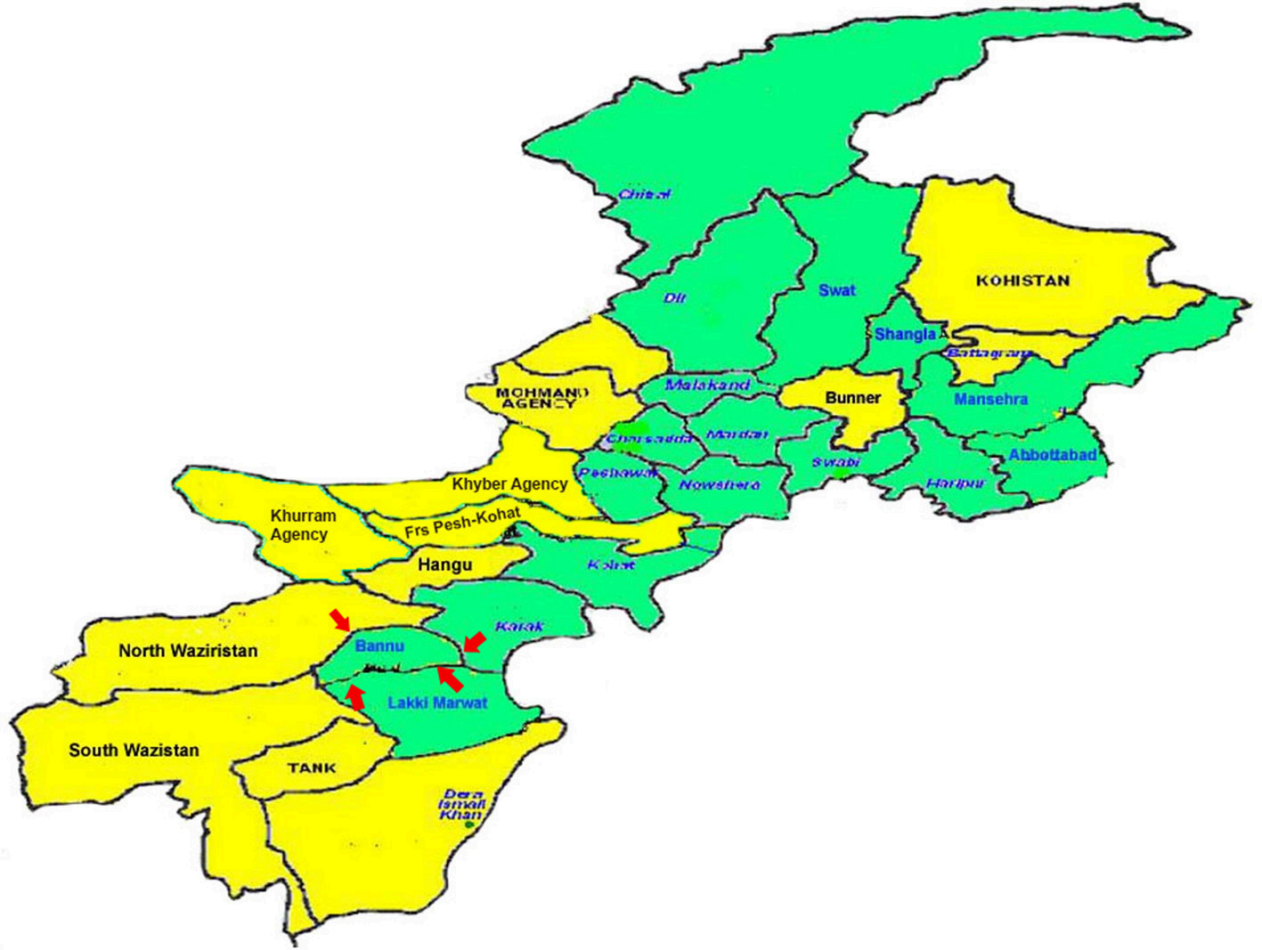
Map of District Bannu.

The district forms a basin drained by the Kurram River and Gambila River (Tochi River) which originating from the hills of Waziristan. The Kurram River, the larger of the two rivers, enters the district from the northwest, near the town of Bannu. From there, it runs first south-east, then south into Lakki Marwat. The Gambila River enters the district about 6 miles (9.7 km) south of the Kurram and flows in the same direction into Lakki Marwat, where the rivers eventually merge (http://kpktribune.com/index.php/en/district-bannu; Figure 1).

### Field interviews

Before starting the ethno-botanical study, contacts were made with various offices (District administration, tourism, culture, and agriculture development, traditional healers and health affairs) to seek permission and to carry out the study. The present study was conducted from March 2014 to March 2016. A total of 130 informants were interviewed (110 females, 20 males) using questionnaire to obtain indigenous knowledge of children diseases in Bannu. The informants were divided into different age groups i.e., 21–30, 31–40, 41–50, 51–60, and above 60 years old. The ethnobotanical data was collected from 13 different villages of district i.e., Shamshi khel, Jandu khel, Sada khel, Shabaz azmat khel, Ismail khel, Surani, Booza khel, Mosa khel, Landidak, Bazar ahmad khan, Goriwala, Mandan, Khojari. Questionnaire (Medicinal Plants Datasheet; Supplementary Table 1) was mainly focused on the ethnobotanical claims and traditional believes of native communities and nearby people. The record questionnaires used included two sections. Section 1 dealt with personal information including age, gender, and educational background. Section 2 was about their practice including the following information: (1) the vernacular name of the plants, (2) plants part/s used for therapeutic value, (3) the way of preparation of various recipes, (4) nature of plant material, (5) relative abundance at the area, (6) habitat of the plant species, (7) mode of application, and (8) medicinal uses of particular plant. Interviews were performed in the local language (Banuchi dialect and Wazirwola). Research articles, books and relevant web pages were also studied with the aim to accumulate data of phytochemical compounds present in the plants.

### Collection, identification, and deposition of medicinal plants

Ethnobotanical data was collected in four seasons (spring, autumn, summer, winter) frequent field visits. In the current study medicinal plants reported by the local informants were identified by vernacular names and collected in the field. Photographs were clipped of the spot and tag the specimen with local name. These specimens were later identified by Taxonomist Dr. Muhammad Zafar, Dr. Mushtaq Ahmad, and Dr. Mir Ajab Khan Department of Plant Sciences Quaid-i-Azam University, Islamabad. The plant name and family has been validated through The International Plant Name Index (IPNI). The collected plant specimens were dried and preserved processed as per routine herbarium techniques recommended by Jain and Rao (1977). The Voucher specimens of each plant were deposited in the herbarium of Department of Plant sciences Quaid-i-Azam University, Islamabad. Plant habit consisted of species characteristics such as herb, shrub and trees that were gathered from the available literature (Ali and Qaiser, 2011; **Table 2**).

### Ailment categories

Based on the information obtained from the informants and non-specialist informants, all the reported ailments were characterized into 14 children categories. These categories includes treating gastrointestinal diseases, respiratory disorders, cardiovascular diseases, rheumatic diseases, ear nose throat (ENT), eye infection, dermatological problems, liver disorders, urinary problems, antidote, dental problems, kidney problems, fever and circulatory diseases.

### Data analysis

The ethno-botanical data were analyzed using Microsoft Office Excel spreadsheet (2010). The species were listed in alphabetical order by scientific name, family, common name, local name, habit, plants part/s used, remedies, mode of utilization, route of application, diseases treated. The data collected through informants were analyzed using different quantitative indices like Use value (UV), Factor of informant consensus (FIC), and Relative frequency of citation (RFC).

### Use value (UV)

The relative importance of a locally known species is calculated by use value (Phillips et al., 1994). UV = ΣUi/ni, Where “Ui” is the number of use-reports cited by each informant for a given species and “ni” is the total number of informants which interviewed for a specific plant species. The Use values are high when there are many use-reports for a plant, which indicate the important plants. When there is few use reports, the Use values approach zero (0). UV does not differentiate whether a plant is used for a single or multiple purposes (Musa et al., 2011).

### Factor of informant consensus (FIC)

Factor of informant consensus (FIC) was used to test the homogeneity of knowledge of medicinal plants. Before analysis, ailments were broadly classified into various categories. According to Heinrich et al. (1998) the FIC was calculated as FIC = Nur – Nt/Nur – 1. FIC values are low (near 0), if plants are selected randomly or there is no exchange of information about their use among local informants. FIC approaches (1) when there is a well-defined selection criterion in the community and/or the information is exchanged between informants (Sharma et al., 2012).

### Frequency citation (FC) and relative frequency citation (RFC)

The FC of the species of plants used in the present study was calculated using the formula: Fc = (Number of times particular species was mentioned/total number of times that all species were mentioned) × 100. The relative frequency citation (RFC) index was calculated as described earlier (Tardio and Pardo-De-Santayana, 2008), using the following formula: RFC = FC/N (0 < RFC < 1). This index is obtained by dividing the number of informants citing a useful species FC or frequency of citation by the total number of informants in the survey (N). RFC value varies from 0 (when nobody refers to a plant as a useful one) to 1(when all the informants mention it as useful).

## Results

### Demographic data of local inhabitants

Total 130 interviews including 110 females and 10 males were conducted in the present study for children diseases. The informants were divided into five different age groups starting from 20 to 60+ years. Most of the informants were females (age 60 years and above) as children disorders information are mostly confined to women in the study area. In these informants 57.7% were illiterate (Tables 1, 2).

**Table 1:** Demographic characteristics of informants *N* = 130.

**Demographical characteristics**	**Number**	**Percentage %**	**Education**
**AGE**			
20–30	15	11.5	Higher level
31–40	20	15.4	Middle
41–50	20	15.4	Primary
51–60	35	27	Illiterate
Above 60	40	30.7	Illiterate
**GENDER**			
Male	10	7.6	
Females	120	92.3	

**Table 2:** Medicinal plants of District Bannu used by local inhabitants for Children diseases.

**Scientific name voucher number**	**Family**	**Common name**	**Local name**	**Habit**	**Part used**	**Remedies**	**Mode of utilization**	**Route of application**	**Disease treated**	FC^*^	RFC^*^	UR^*^	UV^*^
*Acacia nilotica* H.Karst. S-ISL-50	*Leguminosae*	Gum arabic tree	Kikar	Tree	Flower	Flower is boiled in mustard oil dropped 2–3 drops of oil in ear	Raw form	Ear drop	Earache	20	0.15	1	0.05
*Aerva javanica* Juss. S-ISL-15	Amaranthaceae	Desert cotton	Spairai	Herb	Arial parts	Half cup of infusion given before lunch time	Infusion	Oral	Stomach disorders	4	0.03	1	0.25
*Allium cepa* L. S-ISL-12	Liliaceae	Onion	Piyaz	Herb	Bulb	(1) Bulb is heated in cooking oil and applied on boils for whole night (2) 2–3 drops of extract dropped in ear (3) Put lukewarm bulb on eye	Raw form Extract	Topical Ear drop Topical	Boil maturation Earache Eye infection	34	0.26	3	0.08
*Allium sativum* L. S-ISL-4	Alliaceae	Garlic	Ouxa	Herb	Bulb	(1) Bulb is heated in mustard oil for 5–7 min the bulb color is become changed and dropped the oil in ear (2) 2 spoon of extract is given with a glass of milk once a day	Raw form Extract	Ear drop Oral	Earache Cough Tuberculosis	15	0.11	3	0.2
*Azadirachta indica* A.Juss. S-ISL-6	Meliaceae	Margosa tree	Neem	Tree	Leaves Bark	(1) Leaves are wrapped around wounds (2) 1–2 drops of bark decoction dropped in ear and nose (3) Olive oil mixed with bark decoction and massage on affected area (4) Half cup of bark decoction given orally	Raw form Decoction	Topical Ear drop Nasal drop Topical Oral	Wounds Nasal infection Earache Scabies Intestinal worms	16	0.12	5	0.31
*Boerhavia diffusa* Engelm. & A.Gray S-ISL-22	Nyctaginaceae	Hogweed	Pndrosh	Herb	Root	Roots are used by spiritual healers. From roots a necklace is made which worn by patients. As the disease is cured, necklace becomes longer and the patient is comforted when the necklace length reaches the navel	Raw form		Hepatitis	51	0.39	1	0.01
*Calotropis procera* (Aiton) W. T. Ation S-ISL-1	Apocynaceae	Rubber brush	Spalmka	Shrub	Latex Leaves	(1) Latex is applied on boils and region of insects bite at night (2) 2–3 leaves heated in water and then applied on affected area	Raw form	Topical	Boil maturation Insect bite Bone ache	45	0.34	3	0.06
*Cannabis sativa* L. S-ISL-2	Cannabaceae	Cannabis	Bhangy	Herb	Whole plant	Half cup of decoction is given with black pepper	Decoction	Oral	Pertussis	7	0.05	1	0.14
*Cassia fistula* Herbb. ex Oliv. S-ISL-10	Leguminosae	Indian laburnum.	Gard nail	Tree	Fruit Flower	(1) Small amount of fruit with water is given to children (2) half spoon of fruit mixed in mother milk and given to infants (3) fruit powder with water is given to children (4) 2 spoon of flower decoction mixed with sugar and given	Raw form Powder Decoction	Oral	Abdominal pain ConstipationCough Diphtheria Hepatitis	48	0.36	5	0.10
*Citrullus colocynthis* (L.) Schrad S-ISL-19	Cucurbitaceae	Bitter cucumber	Margona	Herb	Seed Fruit	(1) 1–2 seeds are given (2) Dried fruit ground to make powder and mixed with Trachyspermum copticum seed powder then made paste. Make small tablets and given	Raw form Powder	Oral	Diabetes Dysentery Constipation Intestinal worms	38	0.29	4	0.10
*Citrullus lanatus* (Thunb.) Mansf.S-ISL-18	*Cucurbitaceae*	Water melon	Indwara	Herb	Fruit	Fruit is given orally	Raw form	Oral	Cough Hepatitis	29	0.22	2	0.06
*Citrus limon* (L.) Burm.f. S-ISL-21	Rutaceae	Lemon	Lemboo	Shrub	Fruit	2–3 spoon of juice given with a glass of water twice a day	Juice	Oral	Urine problems Vomiting Kidney stone	31	0.23	3	0.09
*Citrus sinensis* Pers. S-ISL-30	Rutaceae	Sweet orange	Malta	Tree	Peel	Half cup of decoction given once a day	Decoction	Oral	Kidney stone *Renal failure*	14	0.10	1	0.07
*Coriandrum sativum* L. S-ISL-34	Apiaceae	Coriander	Dhanrya	Herb	Leaves Seed	(1) 3–6 spoon of infusion is given at lunch time (2) leaves are grinded and mixed in mother milk and dropped in eye (3) small amount of cooked seed is given before dinner	Infusion Raw form	Oral	Dysentery Eye infection Vomiting	19	0.14	3	0.15
*Cucumis melo* Blanco S-ISL-37	*Cucurbitaceae*	Melon	Khrboza	Herb	Peel Fruit	(1) Dried peel is ground to powder and given with honey (2) Fruit is given twice a day	Powder Raw form	Oral	Kidney stone Constipation	22	0.16	2	0.09
*Dalbergia sissoo* Roxb. S-ISL-80	Leguminosae	Indian rose wood	Sawa	Tree	Leaves	Fresh leaves are crushed and rubbed on affected area	Paste	Topical	Scabies Wounds	4	0.03	2	0.5
*Dodonaea viscosa* Royen ex Blume S-ISL-29	Sapindaceae	Hopbush	Sanatha	Shrub	Leaves	Leaves are burnt to obtained ash and mixed with mustard oil then massage on affected area	Ash	Topical	Scabies	5	0.03	1	0.2
*Ficus benghalensis* L. S-ISL-24	Moraceae	Banyan tree	Barh	Tree	Leaves	Ash is sprinkle on wounds	Ash	Topical	Wounds	7	0.05	1	0.14
*Ficus carica* L. S-ISL-16	Moraceae	Fig	Anjeer	Tree	Fruit	4–5 Fruits are given twice a day	Raw form	Oral	Cough Constipation	12	0.09	2	0.16
*Ficus religiosa* Forssk S-ISL-8	Moraceae	Sacred fig tree	Peepal	Tree	Leaves	Leaves are burnt and ash is mixed in water and given	Ash	Oral	Vomiting	8	0.061	1	0.12
*Foeniculum vulgare* Hill. S-ISL-42	Apiaceae	Fennel	Kalwo	Herb	Fruit	(1) Small amount of cooked fruit ground into powder mixed in milk and given to infants (2) half cup of decoction is given	Powder Decoction	Oral	Bellyache Dysentery Indigestion Urinary disorders	19	0.14	4	0.21
*Helianthus annuus L*. S-ISL-44	Asteraceae	Sun flower	Umar gul	Shrub	Leaves Seed	(1) Leaves are heated in water and then rubbed on wounds (2) Seed oil is used for cooking purpose	Raw form Oil	Tropical Oral	Cough, Wounds Irregular heart problem, Heart valve problem	12	0.09	3	0.25
*Jasminum officinale* L. S-ISL-48	Oleaceae	Jasmine	Chmbeli	Shrub	Leaves	2 spoons of decoction is given orally	Decoction	Oral	Toothache	4	0.03	1	0.25
*Melia azadirachta L*. S-ISL-47	Meliaceae	Chinaberry tree	Makanra	Tree	Leaves	1 cup of decoction given before breakfast	Decoction	Oral	Diabetes	19	0.14	1	0.05
*Mentha spicata L*. S-ISL-62	Lamiaceae	Mint	Putna	Herb	Leaves	(1) 1 spoon of powder mixed in one cup of green tea and given (2) 3–4 drops of decoction dropped in ear and nose (3) half cup decoction is given once a day	Powder Decoction	Oral Ear drop Nasal drop Oral	Vomiting Diarrhea Earache Nasal infection Intestinal worms, Hepatitis	25	0.19	6	0.24
*Momordica charantia L*. S-ISL-76	*Cucurbitaceae*	Bitter gourd	Karaila	Herb	Fruit Seed	(1) Fruit is rubbed on wounds (2) Seed ground to powder then one teaspoon is given with sugar	Raw form Powder	Topical Oral	Wounds Intestinal worms	2	0.01	2	1
*Ocimum* basilicum L. S-ISL-98	Lamiaceae	Basil	Babrye	Herb	Leaves	(1) Fresh leaves are crushed and dropped in ear (2) Decoction is used for gargling	Raw form Decoction	Ear drop Oral	Earache Toothache Cough Fever	15	0.11	4	0.26
*Oryza sativa* Hochst. ex Steud S-ISL-33	Poaceae	Rice	Wareejy	Grass	Seed	1 kg of seed and 2 cup of bark powder of Acacia nilotica mixed in clay pot and keep in sunshine for 11 days, seed color become red and ground to powder which given with sugar at night	Powder	Oral	Urine problems	3	0.02	1	0.33
*Peganum harmala* L. S-ISL-66	*Zygophyllaceae*	Wild rue	Spalani	Herb	Whole plant Seed	(1) Decoction of whole plant is used as gargling (2) Seeds are given with water once a day (3) whole plant dipped in mustard oil and massage it on child body for 1 month	Decoction Raw form	Oral	Sour throat Abdominal pain Hepatitis	41	0.31	3	0.07
*Phoenix dactylifera* L. S-ISL-54	*Arecaceae*	Dates	Khajoor	Tree	Leaves Fruit	(1) 2 spoon of decoction given at night (2) 6–7 fruits are given	Decoction Raw form	Oral	Kidney stone Mace	7	0.05	2	0.28
*Phyla nodiflora* (L.) Greene S-ISL-103	Verbenaceae	Cape weed	Bakanra	Herb	Seed	2 spoon of powder given with Syzygium cumini juice twice a day	Powder	Oral	Diabetes	1	0.007	1	1
*Piper longum* Blume S-ISL-91	Piperaceae	Long pepper	Lawang	Herb	Fruit	(1) 1–2 fruits keep in teeth cavity (2) 2 spoon of decoction given	Raw form Decoction	Oral	Toothache	22	0.16	1	0.04
*Piper nigrum* Beyr. ex Kunth S-ISL-96	Piperaceae	Black pepper	Toor march	Herb	Seed	(1) 1 tea spoon of powder sprinkle on boiled egg and given at night (2) Half tea spoon of powder mixed with honey and given (3) 2–3 spoon of decoction is given	Powder Decoction	Oral	Cough, Flu complication Diarrhea Toothache	39	0.3	4	0.10
*Plantago ovata* Phil. S-ISL-101	Plantaginaceae	Psyllium	Ispaghol	Herb	Seed	Seed and husk soaked in water	Raw form	Oral	Dysentery Constipation	44	0.33	2	0.04
*Psidium guajava* L. S-ISL-110	Myrtaceae	Guava	Amrood	Tree	Leaves Fruit	(1) Decoction is used as a gargling (2) Fruit is given orally	Decoction Raw form	Oral	Toothache Constipation	12	0.09	2	0.16
*Punica granatum* L. S-ISL-69	*Lythraceae*	Pomegranate	Anar	Shrub	Peel Fruit	(1) Mother milk heat up in the peel, when the milk color become yellowish then given to infants (2) Fruits are given or squeezed to obtained juice and given twice a day (3) 2 drops of juice dropped in eye	Raw form Juice	Oral Eye drop	Cough, Flu complication Dysentery Vomiting Hepatitis Eye infection	21	0.16	6	0.28
*Pyrus malus L*. S-ISL-75	Rosaceae	Apple	Saib	Tree	Fruit	(1) Fruit is given as they needed (2) Fruit is heated in 2 glass of water with sugar until the water become half and this recipe called Murabba in local language and given	Raw form	Oral	Stomachache Cough Dysentery Vomiting	5	0.03	4	0.8
*Raphanus sativus L*. S-ISL-72	Brassicaceae	Reddish	Molye	Herb	Root	Half cup of decoction is given with sugar before breakfast	Decoction	Oral	Kidney stone Hepatitis	2	0.01	2	1
*Ricinus communis* L. S-ISL-85	Euphorbiaceae	Castor oil plant	Randa	Shrub	Leaves Seed	(1) Leaves are heated in water then dasi ghee is applied on the leaves and wrapped around affected area (2) 1 spoon of seed oil mixed in green tea and given (3) half spoon of oil mixed with mother milk and given to infants	Raw form Oil	Oral	Bone ache Joint pain Wounds Abdominal pain	30	0.23	3	0.1
*Rosa indica* L. S-ISL-94	Rosaseae	Rose	Gulab	Shrub	Flower	(1) 2–3 drops of distilled essence (Arq) dropped in eyes (2) 2 Flowers are kept in half cup of olive oil for 1 week then oil is dropped in ear (3) Oil is rubbed over swelled eye and gums	Raw form	Eye drop Ear drop Tropical	Eye infection Earache Toothache Gum swelling	9	0.06	3	0.3
*Rubus occidentalis* L. S-ISL-99	*Rosaceae*	Black raspberry	Toot	Tree	Fruit Root Leaves bark	(1) Fruits are eaten or become squeeze to obtained juice and given (2) Half cup of decoction mixed with sugar and given (3) Decoction of leaves used for gargling	Raw form Decoction	Oral	Cough Diphtheria Toothache	20	0.15	3	0.15
*Saccharum officinarum* L. S-ISL-100	Poaceae	Sugar cane	Gana	Grass	Stem	One glass of juice given twice a day	Juice	Oral	Hepatitis Urine problems	5	0.02	1	0.3
*Saccharum bengalense* Retz. S-ISL-112	Poaceae	Munj sweet cane	Kana	Grass	Leaves	Half cup of decoction given at night	Decoction	Oral	Urine problem	3	0.03	2	0.4
*Sesamum indicum L*. S-ISL-118	Pedaliacae	Sesame plant	Kunjaly	Herb	Seed	(1) Oil massage on affected area (2) Seeds are given with gurr twice a day	Oil	Tropical Oral	Scabies Urine problems Bladder problem	15	0.11	2	0.13
*Solanum lycopersicum* Blanco S-ISL-59	Solanaceae	Tomato	Tamatar	Herb	Fruit	2–3 fruits are given orally at lunch time	Raw form	Oral	Hepatitis, Anemia	8	0.06	2	0.25
*Solanum surattense* Burm.f. S-ISL-67	Solanaceae	Yellow berried nightshade	Kharyarmargona	Herb	Fruit	(1) Half teaspoon of Powder inhaled (2) half cup of decoction is given before lunch time	Powder Decoction	Inhaled Oral	Flu complication Liver problems	11	0.08	2	0.18
*Syzygium cumini* (L.) Skeels S-ISL-67	Myrtaceae	Java plum	Jaman	Tree	Fruit	Fruits are given orally trice a day	Raw form	Oral	Diabetes, Dysentery, Liver problem	29	0.22	2	0.06
*Tamarix aphylla* (L.) H.Karst. S-ISL-28	Tamaricaceae	Athel tamarisk	Ghazz	Tree	Leaves	1 cup of decoction with 2 spoon of honey given once a day	Decoction	Oral	Dysentery Cough	9	0.06	2	0.2
*Thuja occidentalis L*. S-ISL-95	Cupressaceae	Eastern white cedar	Sarwa	Tree	Fruit	Half cup of decoction is given with black pepper once a day	Decoction	Oral	Dysentery Constipation Urine problem	8	0.06	3	0.3
*Trachyspermum copticum* Link S-ISL-82	Apiaceae	Carom	Kabli sparai	Herb	Seed	3–4 drops of infusion is given to infants	Infusion	Oral	Vomiting	12	0.09	1	0.08
*Tribulus terrestris* L. S-ISL-107	Zygophyllaceae	Bullhead	Kunda	Herb	Whole plant	Half cup of decoction is given once a day	Decoction	Oral	Kidney stone	4	0.03	1	0.2
*Trigonella foenum-graecum Sm* S-ISL-111	Leguminosae	Fenugreek	Malkhoj	Herb	Seed	(1) Dried seed ground to obtained powder then made paste and applied on wounds (2) 1 tea spoon of powder with sugar given to children	Powder	Tropical Oral	Wounds Dysentery	18	0.13	2	0.1
*Withania coagulans* Dunal S-ISL-120	Solanaceae	Indian rennet	Khmzor	Herb	Seed	2, 3 seeds socked in mother milk and after 1 h milk is given to infants	Raw form	Oral	Abdominal pain, Gas trouble	32	0.24	2	0.06
*Ziziphus jujube* Lam. S-ISL-40	Rhamnaceae	Jujube	Baira	Tree	Leaves Fruit	(1) Fruit is given orally (2) Leaves are crushed and add small piece of washing soap then applied on affected area	Raw form Paste	Oral Tropical	Diabetes Boils maturation	10	0.07	2	0.2
*Ziziphus nummularia* (Burm.f.) Wight & Arn S-ISL-57	Rhamnaceae	Jharber	Kankarh	Shrub	Fruit Leaves	(1) 4–7 fruits eaten before breakfast for 3 days, (2) 2–3 leaves are chewed	Raw form		Heart problems, Hemophilia, Toothache	4	0.03	2	0.5

FC^*^, Frequency of citation; RFC^*^, Relative Frequency of Citation; UR^*^, Use Report; UV^*^, Use Value.

### Most used families and their family importance value (FIV)

**Figure 2:**
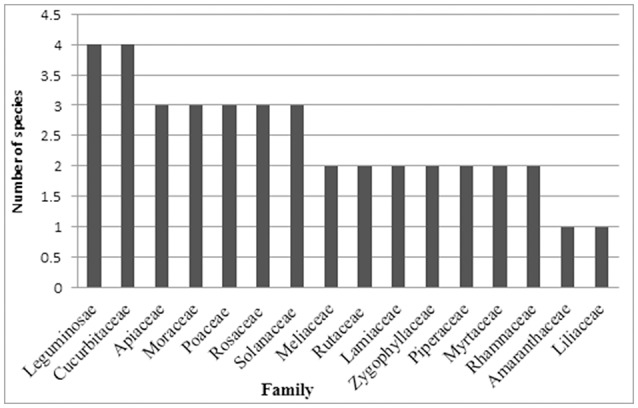
Families used in children diseases.

The present study reported the uses of 55 plant species belonging to 49 genera and 32 different families that are commonly used by local inhabitants to treat various children diseases (Table 2). The largest number of specie belong to Leguminosae and Cucurbitaceae families (4 species each) followed by Apiaceae, Moraceae, Poaceae, Rosaceae, and Solanaceae (3 species each), Meliaceae, Rutaceae, Lamiaceae, Zygophyllaceae, Piperaceae, Myrtaceae, and Rhamnaceae (2 species each), and the remaining 18 families were represented by one plant species each (Table 3; Figure 2). The highest number of medicinal plant species from these families may be due to their wider distribution and richness in the study area. The present study supported Kadir et al. (2012) who reported Leguminosae family as the dominant family (Table 3; Figure 2).

**Table 3:** Families used in children diseases with their FIV values.

**S. no**.	**Family**	**Species**	FIV^*^
1	Leguminosae	4	7.27
2	Cucurbitaceae	4	7.23
3	Apiaceae	3	5.45
4	Moraceae	3	5.45
5	Poaceae	3	5.45
6	Rosaceae	3	5.45
7	Solanaceae	3	5.45
8	Meliaceae	2	3.63
9	Rutaceae	2	3.63
10	Lamiaceae	2	3.63
11	Zygophyllaceae	2	3.63
12	Piperaceae	2	3.63
13	Myrtaceae	2	3.63
14	Rhamnaceae	2	3.63
15	Amaranthaceae	1	1.81
16	Liliaceae	1	1.81
17	Alliaceae	1	1.81
18	Nyctaginaceae	1	1.81
19	Apocynaceae	1	1.81
20	Cannabaceae	1	1.81
21	Sapindaceae	1	1.81
22	Asteraceae	1	1.81
23	Oleaceae	1	1.81
24	Arecaceae	1	1.81
25	Verbenaceae	1	1.81
26	Plantaginaceae	1	1.81
27	Lythraceae	1	1.81
28	Brassicaceae	1	1.81
29	Euphorbiaceae	1	1.81
30	Pedaliacae	1	1.81
31	Tamaricaceae	1	1.81
32	Cupressaceae	1	1.81

FIV^*^, Family importance value.

### Life form of plants used in children diseases

**Figure 3:**
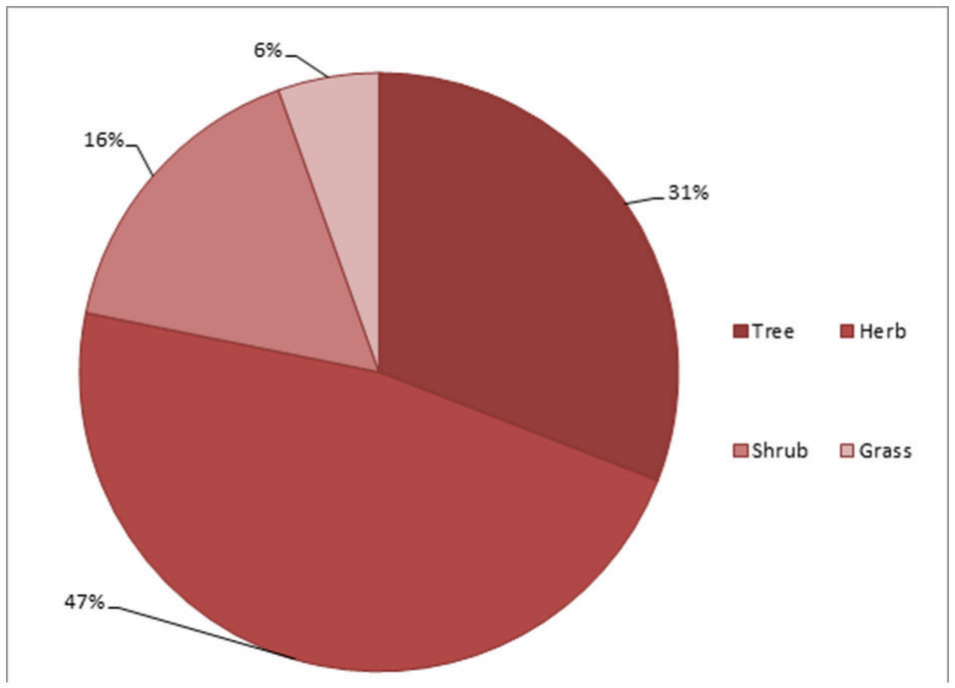
Life form of medicinal plants used in children diseases.

Herbs (47%) were the primary source of indigenous medicine among 55 species, followed by trees (31%), shrubs (16%), and grasses (6%) (Figure 3). Herbs were mostly used by local inhabitants due to greater availability and access of such herbaceous plants in the study area (Figure 3).

### Plant parts used in herbal medicines

**Figure 4:**
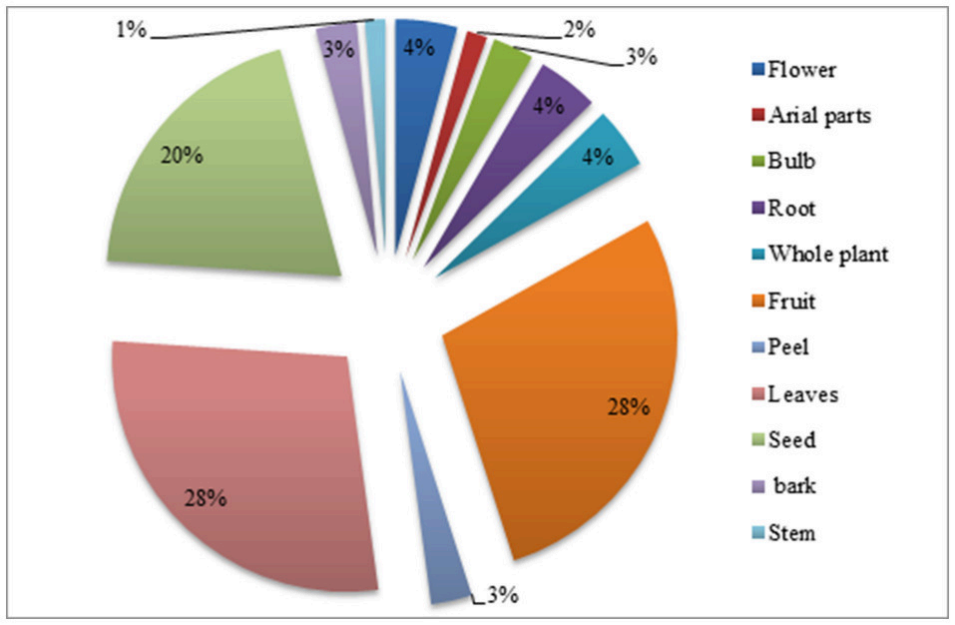
Percentage of plant parts used in children diseases.

In different parts of these plants numerous secondary metabolites were present that are very important that fight against plant's pathological stress and can be used by local peoples to cure variety of diseases (Croteau et al., 2000; Shah et al., 2013, 2014a,b). Fruit and leaves were the most frequently used parts (28%), followed by seeds (20%), flower, whole plant, root (4% each), bark, peel, bulb (3% each) and stem, arial parts correspond to 3% each (Figure 4). Thus, fruit and leaves were largely used in the study area. There were no specific documented literatures on children diseases; therefore generally available ethno-botanical literature was used for comparison. The results supported the previous studies by Mahishi et al. (2005), Abo et al. (2008), González et al. (2010), Telefo et al. (2011), and Kadir et al. (2012) that leaves were dominant part used. Collection of leaves and then using them as medication is easy as compared to flowers, fruits and roots (Giday et al., 2009; Telefo et al., 2011). The digging out roots might be the cause of loss of the plant and putting the species in a susceptible condition (Figure 4) (Poffenberger et al., 1992; Martínez et al., 2000; Zheng and Xing, 2009; Rehecho et al., 2011).

### Mode of utilization and route of application of medicinal plants

**Figure 5:**
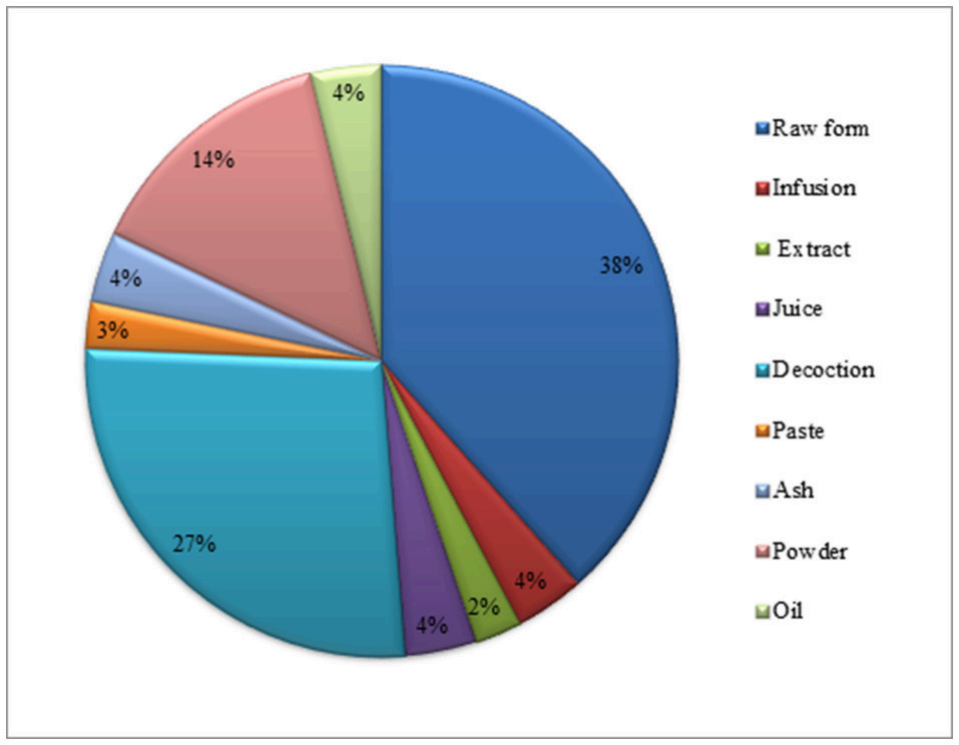
Percentage of mode of utilization for children diseases.

**Figure 6:**
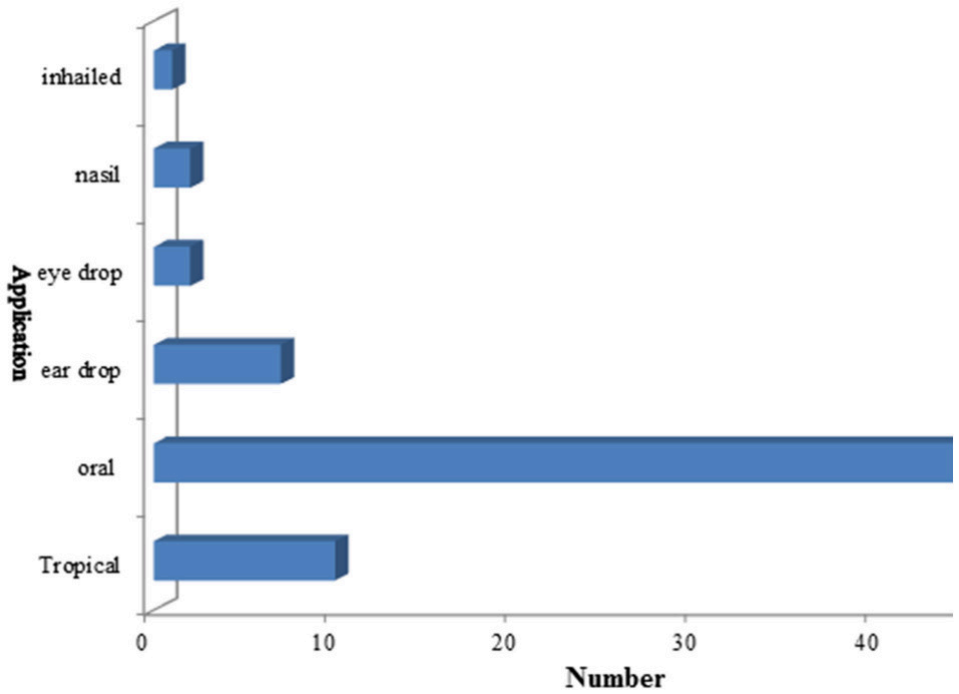
Route of application of children disorders.

Herbal preparations were used in different forms like decoction, powder, extract, juice, infusion, paste, raw form, and cooked. Raw form utilization of these herbal plants for child diseases were the most common method (38%), followed by decoction (27%), powder (14%), oil, ash, infusion, and juice (4% each), paste (3%), and extract (2%) (Figure 5). The main route of application for herbal therapies was oral (68.5%), followed by the topical (14%), ear drop (10%), eye drop, nasal (3.5% each), and inhaled remedies (1.5%) (Figure 6). Oral mode of administration is preferred route all over the world (Figures 5, 6).

### Use categories in children diseases

**Figure 7:**
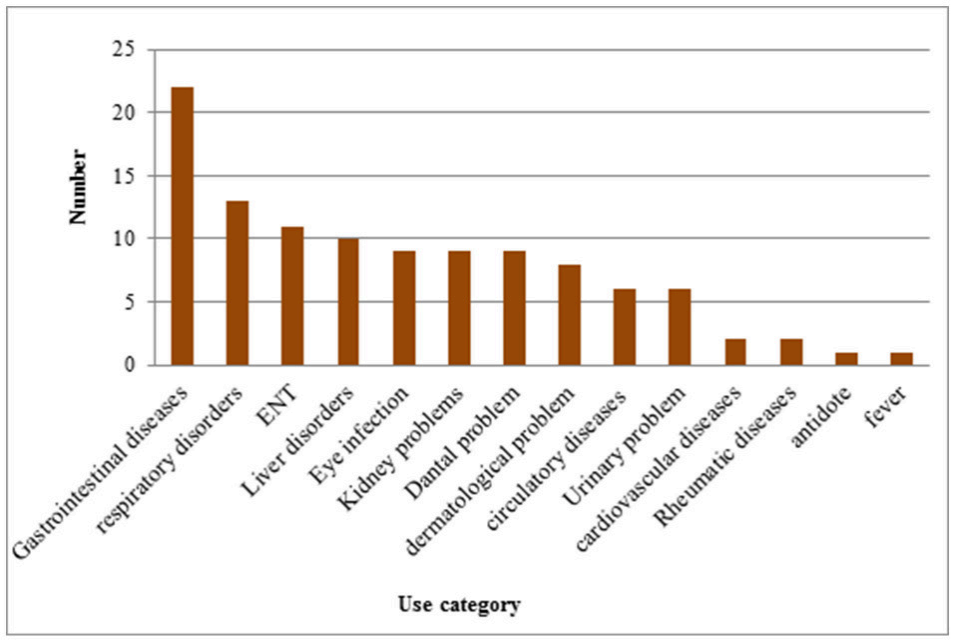
Medicinal plants use categories for children diseases.

Local inhabitants used medicinal plants species to cure different ailments. These ailments were grouped into 14 broad disease categories including gastrointestinal diseases, respiratory disorders, ear nose throat (ENT), liver disorders, eye infection, kidney problems, dental problem, dermatological problem, antidote, circulatory diseases, fever, urinary problem, cardiovascular diseases and rheumatic diseases. In the study area the highest number of plant species were used in the treatment of gastrointestinal diseases (39 species) followed by respiratory disorders (16 species), ear nose throat (ENT) (13 species), liver disorders (11 species), eye infection, kidney and dental problem (10 species), dermatological problem (9 species), circulatory and urinary problem (7 species), cardiovascular and rheumatic diseases (3 species) and antidote (1 species) (Figure 7; Table 4). Ethno pharmacological studies have shown that in most parts of the world, the gastrointestinal disorder is the first use category (Heinrich et al., 1998; Miraldi et al., 2001; Ghorbani, 2005; Ghorbani et al., 2011; Mosaddegh et al., 2012). In the study area due to poor dietary environments and risky drinking water, gastrointestinal is one of the most common problem. The present study strongly agreed with the previous result of Bibi et al. (2014) and Sadeghi et al. (2014) that reported gastrointestinal and respiratory disorders as dominant problems in their studies. Ullah et al. (2013) showed a similar study in district Wana province of Khyber Pakhtunkhwa Pakistan using medicinal plants for the cure of gastrointestinal complaints (Figure 7).

**Table 4:** Factor of informant consensus (FIC) and use report (UR) for categorized ailments.

**S no**.	**Ailment categories**	UR^*^	**Number of taxa**	FIC^*^
1	Cardiovascular diseases (Hemophilia and heart problems)	3	2	0.5
2	Rheumatic diseases (Bone ache, Joint pain)	3	2	0.5
3	Gastrointestinal diseases (Stomach disorders, Intestinal worms, dysentery, Constipation Vomiting, Diarrhea, Indigestion)	39	22	0.45
4	Respiratory disorders(cough, Diphtheria, Pertussis)	16	13	0.2
5	ENT (ear, nose, throat disorders)	13	11	0.16
6	Circulatory diseases (Diabetes, Anemia)	7	6	0.16
7	Urinary problem (Urine problems, Bladder problem)	7	6	0.16
8	Dermatological problem (scabies, wounds)	9	8	0.12
9	Eye infection (eye swelling, inflammation)	10	9	0.11
10	Kidney problem (kidney stone, Renal failure)	10	9	0.11
11	Dental problem (toothache, gum swelling)	10	9	0.11
12	Liver disorders (Hepatitis)	11	10	0.1
13	Antidote (insect bites)	1	1	0
14	Fever	1	1	0

UR^*^, Use report; FIC^*^ factor of informant consensus.

### Use value of medicinal plants

The highest use value reported in the present study was 1, and the lowest value was 0.01 (Table 2). The highest use value were reported for *Momordica charantia* and *Raphnus sativus* (2 use report/use-value 1 for each), *Phyla nodiflora* (1 use report/use-value 1), *Pyrus malus* (4 use report/use-value 0.8), *Dalbergia sissoo* and *Ziziphus nummularia* (2 use report/use-value 0.5) for each, *Saccharum officinarum* (2 use report/use-value 0.4), *Thuja occidentalis* (3 use report/use-value 0.37), *Rosa indica* (3 use report/use-value 0.33), *Oryza sativa* and *Saccharum bengalense* (1 use report/use-value 0.33 for each) *Azadirachta indica* (5 use report/use-value 0.31), *Punica granatum* (6 use report/use-value 0.28), and smallest use values were reported for *Acacia nilotica* (1 use report/use-value 0.05), *Plantago ovata* (2 use report/use-value 0.04), *Piper longum* (1 use report/use-value 0.04), *Boerhavia diffusa* (1 use report/use-value 0.01). The high use values of plants might be attributable to their wide distribution, making these plants the, first selection for treatment. The present study is the first report of quantitative ethno-botanical data on children disease from Pakistan and to best of our knowledge no efforts have been carried out to document the medicinal flora specifically in this regard globally.

### Factor of informant consensus (FIC)

The Factor of informant consensus (FIC) of medicinal plants in the present study ranges from (0–0.05) (Table 2). Cardiovascular and Rheumatic diseases have highest FIC values (0.5 for each). The second highest value was observed for gastrointestinal diseases (0.45), Respiratory disorders, ear nose throat (ENT) and circulatory diseases (0.16 for each). The zero FIC value was observed for antidote and fever (Table 4). As there was no previous record therefore for quantitative data comparison were used from the available ethno botanical literature of medicinal plants. Present study does not support the results of Ullah et al. (2014) and Bibi et al. (2014) reporting highest FIC value for respiratory problems and antidote while 0.45 gastrointestinal diseases in the present study, respectively. Furthermore, Ullah et al. (2014) also reported exact same FIC value for gastrointestinal diseases (0.45) during ethno-botanical survey of medicinal plants in Lakki Marwat.

### Relative frequency of citation (RFC) and use reports (UR)

**Figure 8:**
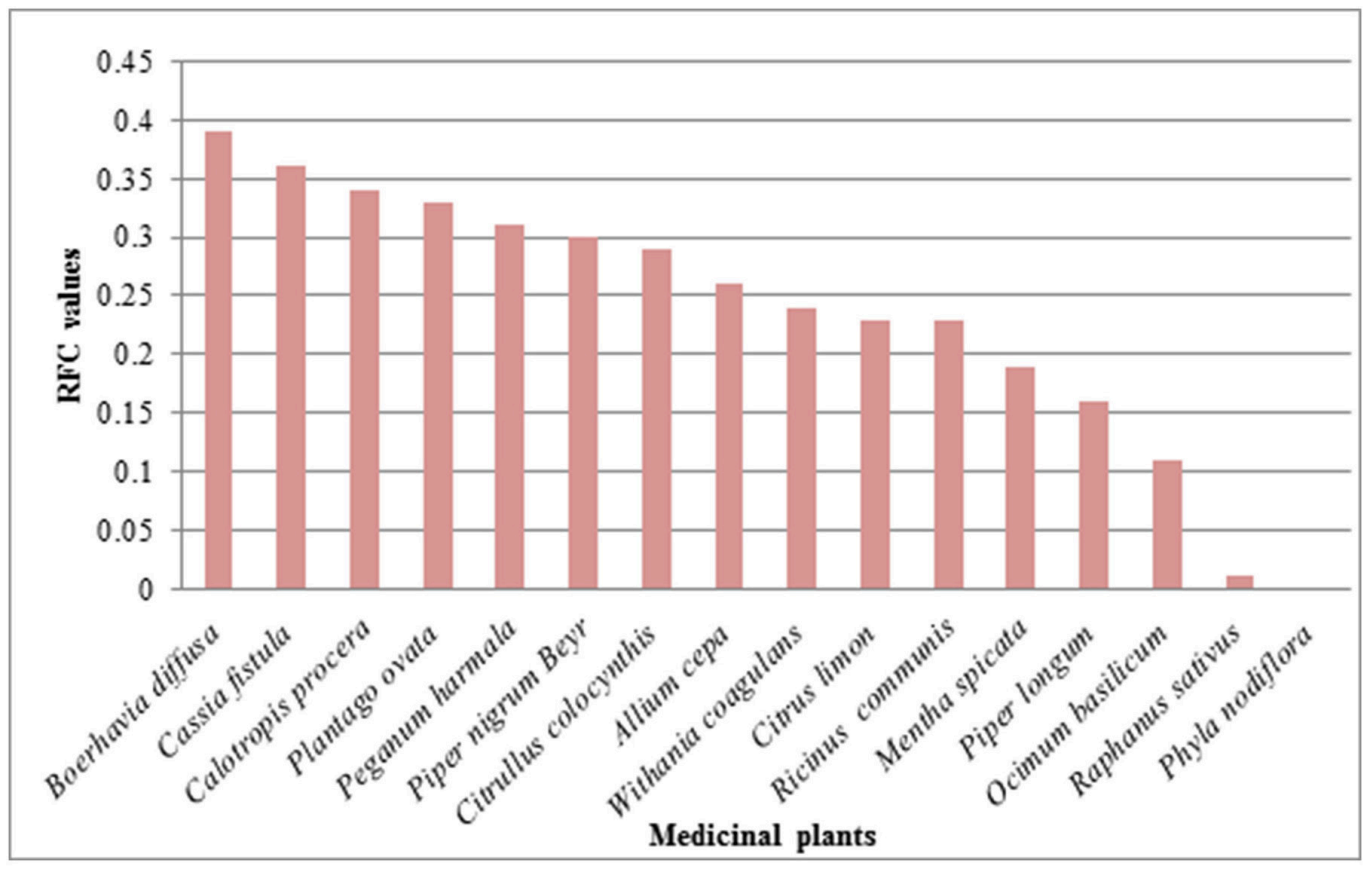
RFC values of medicinal plants used in children disease.

**Figure 9:**
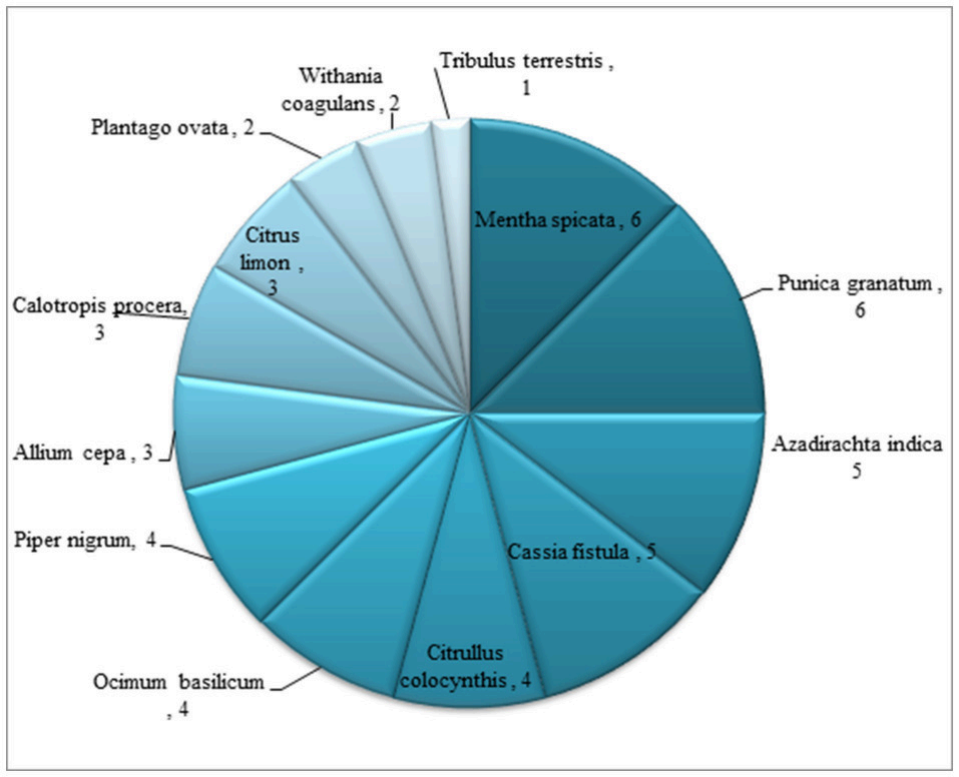
Use reports of medicinal plants used in children diseases.

RFC value varied from 0 to 0.39 in the present study. Maximum RFC value was calculated for species *Boerhavia diffusa* (0.39) followed by *Cassia fistula* (0.36), *Calotropis procera* (0.34), *Plantago ovata* (0.33), *Peganum harmala* (0.31), *Piper nigrum* (0.30), *Citrullus colocynthis* (0.29), *Allium cepa* (0.26), *Withania coagulans* (0.24), *Citrus limon* and *Ricinus communis* (0.23 for each), *Mentha spicata* (0.19), *Piper longum* (0.16), *Ocimum basilicum* (0.11), *Raphanus sativus* (0.01) and *Phyla nodiflora* had zero RFC value (Figure 8; Table 2). Highest RFC values showed that these species are the most popular medicinal plants agreed by the majority of the informants as they are the most popular plants in District Bannu. Species *Mentha spicata* and *Punica granatum* has highest use report value (6 for each) followed by *Azadirachta indica* and *Cassia fistula* (5 each), *Citrullus colocynthis, Ocimum basilicum* and *Piper nigrum* (4 for each), *Allium cepa, Calotropis procera* and *Citrus limon* (3 for each), *Plantago ovata, Withania coagulans* (2 for each) and *Tribulus terrestris* has 1 use report value (Figures 8, 9).

## Discussion

### Medicinal plants and their potential in present study

In children diseases the highest use report in the present study were documented for *Punica granatum, Mentha spicata* (6), *Azadirachta indica*. (5), *Foeniculum vulgare, Cassia fistula, Pyrus malus and Piper nigrum, Ocimum basilicum* (4 for each), *Calotropis procera, Rosa indica, Allium sativum, Allium cepa, Ricinus communis, Citrus limon, and Peganum harmala*, (3 for each), *Withania coagulans, Ziziphus jujube and Plantago ovata* (2 for each). These plants were also reported by others groups for other various human disorders. *Withania coagulans* is used as a blood purifier in hepatic disorders, dermatological problems, dyspepsia, diabetes, and flatulent colic (Panhwar and Abro, 2007), and as a diuretic, for stomach ache and refrigerant (Ullah et al., 2010). *Calotropis procera* is used for backache, skin infections, piles, rheumatism, asthma, cough, and snake, dog, and scorpion bites (Saqib et al., 2014) while it has been used for toothache, abscesses and joint pain by Bhatia et al. (2014). The latex of *Calotropis procera* is also used to cure eczema, snake bites, ringworms, and abdominal cramps, to soften the affected area, and wound healing (Marwat et al., 2008; Abbasi et al., 2010). *Plantago ovata* is mostly used for the treatment of jaundice (Rashid et al., 2012), spermatorrhea, constipation (Marwat et al., 2008), dysentery, diarrhea (Sharma and Kumar, 2007) and hyperacidity (Islam et al., 2011). Allium cepa is important for prevention of heart diseases, boils maturation and insect bite, *Allium sativum*, mostly used for prevention of heart diseases, carminative, boils maturation, earache, Citrus limon used against fungal infections of skin (Ullah et al., 2014). *Sesamum indicum* used for constipation (Noumi and Yomi, 2001). Cucumis melo for constipation (Qureshi, 2012). *Foeniculum vulgare* is used for anemia, jaundice, liver inflammation, Piper nigrum for abdominal pain, and *Ziziphus jujube* for cough (Abbasi et al., 2010). *Ricinus communis* is mostly used for constipation and sedative (Alam et al., 2011).

### Medicinal plant use knowledge in the area

The results of the present study show that the older informants prevails more knowledge than younger ones. The female informants (aged 50 or 60) possessed higher knowledge of medicinal plants used for children's health care. This is not surprising because certain abilities such as medicinal plants knowledge are developed over a life time. A large part of the traditional knowledge is embedded in practices that the people involved in Pfeiffer and Butz (2005) and Guimbo et al. (2011), for example, in rural Niger, female knew more about edible plants than male because they have concern and responsibility for daily food production, whereas men have a better knowledge of fodder and construction (Guimbo et al., 2011). In the present study few plants such as *Areca catechu* and *Myristica fragrans* were not grown in the study area therefore they were purchased from market.

### Threats to medicinal plants in the study area

In the study area many medicinal plants are used and the use of traditional plant knowledge is abundant. The role of medicinal plants in the primary healthcare of the villagers has been reduced because of easier access to modern medicines and changes in their lifestyle. A similar condition was also observed within Huai Labaoya and Samoon Mai Village in Nan Province (Srithi et al., 2009). In Vietnam the development of nurseries for the spread of plants by seeds and stem cuttings on a community level was also effectively practiced (Khanh et al., 2000). Majority of the local people are illiterate especially in the rural areas of the district and the earning sources are only agriculture and livestock. Some of the local inhabitants collect medicinal plants and sell them to the local herb sellers in very low-price and these species are imported to the pharmaceutical companies. Urbanization, over grazing, and uprooting of medicinal plants are severe threats in the study area. These threats increase the risk of plants extinction, therefore proper management strategies should be adopted for a strict control over their protection and safety. The cultivation of medicinal plants and viable use of wild flora should be promoted in the area; thus might help in improving the socioeconomic condition of the local inhabitants.

## Conclusion

The present study provides valuable information about medicinal plants used by local informants to cure children diseases in District Bannu, KPK, Pakistan. 55 medicinal plant species belonging to 49 genera and 32 families were used in the present study. It was first attempt to document ethno-botanical information using quantitative approaches on specific diseases in the study area. The leaves and fruits were mostly used parts (28%), herbs were the most used life form (47%), decoction was the way of administration (27%) and oral ingestion was the main way of application (68.5%) used in herbal medication. The quantitative approaches such as Factor of informant consensus (FIC), Relative Frequency of Citation (RFC), and Use Report (UV) were used in the present study. In children diseases highest use values and FIC values were reported for *Momordica charantia, Raphnus sativus* (1 for each) and Cardiovascular, Rheumatic diseases (0.5 for each), respectively. The results represent useful and long-lasting information about medicinal plants, which may preserve indigenous knowledge of medicinal plants and attract future generations toward traditional healing practices. Due to present study, plants that scored highest FIC, UV values, could be considered as good indicator of prospective plants for discovering new drugs. However there is a gradual loss of traditional knowledge in new generation. Thus it is felt important to document and reconstitute the remainders of the ancient medical practices, which exist in the study area as well as other parts of the region and preserve this knowledge for future generations. The traditional medicine used in the region lacks physiotherapeutic evidence. It is necessary to perform phytochemical and pharmacological studies to explore the potential of such plants herbal drug discovery.

## Author contributions

SS carried out the study and wrote the manuscript; SA, MU, MA, MAh, JH, FM, UF, and AK designed the experiments, did sampling and supervised the work; all authors read and approved the final manuscript.

### Conflict of interest statement

The authors declare that the research was conducted in the absence of any commercial or financial relationships that could be construed as a potential conflict of interest.
