# Supplementary material for: Knowledge of Medicinal Plants for Children Diseases in the Environs of District Bannu, Khyber Pakhtoonkhwa (KPK)
Source: Front Pharmacol. 2017 Jul 17;8:430. doi: 10.3389/fphar.2017.00430 (PMC5511814; doi:10.3389/fphar.2017.00430)
Supplement: Supplementary file 1 [file Table1.DOCX]

**Supplementary Table 1| Ethno botanical Questionnaire**

**Informant information:**

Locality (Village):_______________________

Date: ________________________________

Name of informant_______________________

Age: ___________________________________

Gender: a) Male b) Female

Occupation: ___________________________

Household size: ________________________

Qualification (If any):____________________

Number of earning members: _____________

**B. Information about plants collected:**

Vernacular Name: ________________________

Botanical Name: __________________________

Family: ________________________________

Habit: _________________________________

Habitat: ________________________________

Ethno medicinal Use: Yes/No

If Yes then against which disease? Gynecological or children diseases? ____________

Part of Plant Used for ethno botanical purpose:

a) Root b) Stem c) Leaves d) Fruit e) Flower d) Bark

f) Flower g) Rhizome h) Bulb i) Seed

Mode of utilization:

a) Decoction b) Infusion c) Powder d) Paste e) Cooked F) oral

Mode of application/dosage: _____________________________________________________________________________________________________________________________________________________________________________________________________________________________

Is that plant or its parts available in the market? Yes/ No

If available then at which price per Kg? ______________________
